# N6-Methyladenosine Modification and Its Regulation of Respiratory Viruses

**DOI:** 10.3389/fcell.2021.699997

**Published:** 2021-07-23

**Authors:** Qianyu Feng, Hongwei Zhao, Lili Xu, Zhengde Xie

**Affiliations:** ^1^Beijing Key Laboratory of Paediatric Respiratory Infection Diseases, Key Laboratory of Major Diseases in Children, National Key Discipline of Paediatrics (Capital Medical University), Ministry of Education, National Clinical Research Center for Respiratory Diseases, National Center for Children’s Health, Beijing Paediatric Research Institute, Beijing Children’s Hospital, Capital Medical University, Beijing, China; ^2^Research Unit of Critical Infection in Children, Chinese Academy of Medical Sciences, Beijing, China

**Keywords:** N6-methyladenosine, RNA modification, respiratory syncytial virus, adenovirus, human metapneumovirus, influenza A virus

## Abstract

N6-methyladenosine (m^6^A) is a ubiquitous RNA modification in eukaryotes. It plays important roles in the translocation, stabilization and translation of mRNA. Many recent studies have shown that the dysregulation of m^6^A modification is connected with diseases caused by pathogenic viruses, and studies on the role of m^6^A in virus-host interactions have shown that m^6^A plays a wide range of regulatory roles in the life cycle of viruses. Respiratory viruses are common pathogens that can impose a large disease burden on young children and elderly people. Here, we review the effects of m^6^A modification on respiratory virus replication and life cycle and host immunity against viruses.

## Introduction

RNA posttranscriptional modification is very common in eukaryotes, and more than one hundred types of modifications have been reported to date (Boccaletto et al., 2018). Most of these modifications are deposited on non-coding RNAs, such as ribosomal RNA (rRNA) and transfer RNA (tRNA), which play roles in regulating RNA structure, function, and translation (Cantara et al., 2010; Boccaletto et al., 2018). Among these modifications, m^6^A is the most common modification to poly(a) RNA components, and it is thought to be involved in the processing of messenger RNA (mRNA) (Desrosiers et al., 1974). The m^6^A modification involves a dynamically reversible process that is catalyzed by methyltransferases (writers) and eliminated by erasers. In addition, m^6^A can interact with m^6^A-binding protein (reader) or indirectly modulate the structure of RNA to regulate the interaction between RNA reader proteins (Jia et al., 2011; Liu et al., 2014; Ping et al., 2014). Recent evidence has shown that m^6^A participates in multiple stages of mRNA life, such as RNA folding and structure, maturation, stability, splicing, export, translation, and decay, and it plays an important role in these stages (Liu and Zhang, 2018).

In recent years, increasing numbers of studies have been conducted on the role of the m^6^A modification in different pathogenic viruses, with results revealing its impact on the regulation of the viral life cycle, such as its influence on the expression of specific genes related to the viral life cycle and its possible inhibition or promotion of the replication of various pathogenic viruses (Imam et al., 2020).

With the spread of all kinds of respiratory virus infections worldwide, respiratory virus infections have received increased attention. Respiratory virus infection can cause pneumonia in children, and severe cases can lead to acute respiratory distress syndrome, toxic encephalopathy, heart failure, etc., and in the elderly, it can aggravate underlying lung diseases and be life threatening. Respiratory viruses mainly include influenza virus, respiratory syncytial virus, and adenovirus. Influenza and respiratory syncytial virus cause nearly 300,000 deaths in children under 5 years old every year, while adenovirus and other viruses are associated with high morbidity and mortality rates (Nair et al., 2010; Wang et al., 2020). In particular, several emerging respiratory viruses with potentially epidemic characteristics, such as SARS-CoV-2, which causes coronavirus disease 2019 (COVID-19), threaten the world. The burden of disease caused by respiratory viruses has received increasing attention.

In this paper, the role of the m^6^A modification in the replication of respiratory viruses and its effect on the host immune response are discussed.

### RNA m^6^A Modifications and Protein Factors

The m^6^A modification is regulated by methyltransferase, demethylase and m^6^A RNA-binding proteins. Methyltransferases play roles as “writers” and include methyltransferase-like 3 (METTL3), METTL14, and Wilm’s tumor-associated protein (WTAP). Among these proteins, METTL3 and METTL14 form heterodimers and promote intracellular mRNA methylation (Liu et al., 2014), but only METTL3 has methyltransferase activity, with METTL14 involved in substrate recognition (Śledź and Jinek, 2016; Wang P. et al., 2016; Wang X. et al., 2016). In addition, METTL14 can provide RNA-binding scaffolds, participate in allosteric activation and enhance the catalytic function of METTL3 (Śledź and Jinek, 2016). WTAP drives the m^6^A “writer” complex to the splice site by interacting with the METTL3/METTL14 heterodimer (Liu et al., 2014; Wu et al., 2016). Recently, two other members of the m^6^A methyltransferase complex, RBM15, and its homologous partner, RBM15b, have been shown to recruit the METTL3/METTL4 protein complex to target RNA for selective methylation (Patil et al., 2016).

Demethylation enzymes, including fat mass and obesity protein (FTO) and ALKB homologous 5 (ALKBH5), belong to the family of Fe^2 +^ /alpha-ketoglutarate-dependent dioxygenases and play an “eraser” role in m^6^A methylation (Tan and Gao, 2018). FTO is located in the nucleus and cytoplasm (Jia et al., 2011; Cheung et al., 2013), and ALKBH5 is located in the nucleus (Zheng et al., 2013). FTO regulates the exon splicing of the adipogenic regulator RUNX1T1 by regulating the level of m^6^A near splice sites, thereby regulating differentiation (Zhao et al., 2014). The demethylation activity of ALKBH5 significantly affects mRNA output, RNA metabolism and mRNA processing factor assembly in nuclear speckles (Zheng et al., 2013).

The role of m^6^A RNA-binding protein is as a “reader.” The most well-known readers include the three members of the YTH domain family (YTHDF), YTHDF1, YTHDF2 and YTHDF3, located in the cytoplasm (Dominissini et al., 2012; Wang et al., 2015; Shi et al., 2017). In addition, two other readers are YTHDC proteins, in which YTHDC1 is located in the nucleus and YTHDC2 is located in the cytoplasm (Morohashi et al., 2011; Xu et al., 2014). YTHDF1 directly interacts with the translation initiation factor eukaryotic initiation factor 3 (eIF3) to increase the translation efficiency of mRNA targets (Wang et al., 2015), while YTHDF2 directly recruits the CCR4-NOT dehydrogenase complex to accelerate the degradation of m^6^A -modified RNA (Wang et al., 2014). Previous studies have shown that the N-terminal domain of YTHDF2 facilitates YTHDF2-mRNA complex localization in P-bodies, which are involved in mRNA degradation (Du et al., 2016). YTHDF3 promotes the functions of YTHDF1 and YTHDF2, cooperates with YTHDF1 to promote translation, and cooperates with YTHDF2 to promote mRNA activity attenuation (Li et al., 2017; Shi et al., 2017). YTHDC1 can drive the binding of methylated mRNA targets to serine and arginine rich splicing factor 3 (SRSF3) and nuclear export factor 1 (NXF1) to form mRNA-protein complexes (mRNPs), which together promote nuclear export of RNA (Roundtree et al., 2017). In addition, YTHDC1 is competitively bound by SRSF3 and SRSF10 to regulate RNA splicing (Xiao et al., 2016). YTHDC2 interacts with small ribosomal subunits and XRN1 to affect RNA translation and degradation, respectively (Hsu et al., 2017; Wojtas et al., 2017). In addition to members of the YTH family, other proteins have been reported to bind to m^6^A. The combination of an m^6^A-modified 5′UTR and eIF3 complex can directly recruit the 43S preinitiation complex to the 5′UTR of the mRNA, thereby stimulating translation initiation (Meyer et al., 2015). Splicing and microRNA maturation is regulated by the binding of hnRNPA2/B1 to m^6^A-modified RNA (Alarcón et al., 2015). Under normal and stress conditions, insulin-like growth factor 2 mRNA-binding proteins (IGF2BPs) promote the stability and storage of their target mRNAs in an m^6^A-dependent manner, thereby affecting gene expression output (Huang et al., 2018).

### The Role of m^6^A During Respiratory Virus Infections

#### m^6^A and Adenovirus

An adenovirus (AdV) is a DNA virus that relies on the processing of host RNA (Price et al., 2020). It uses cellular RNA polymerase II and spliceosome mechanisms to produce early and late genes from two DNA strands to produce mature mRNA (Sommer et al., 1976). Price et al. (2020) found that after adenovirus infection of A549 cells, the expression levels of other interacting enzymes did not change significantly, except for a slight increase in the levels of FTO and ALKBH5, and the host proteins were concentrated at the sites of early viral RNA synthesis. Through indirect immunofluorescence microscopy, it has been observed that some writer proteins, such as METTL3, METTL14, and WTAP, and reader protein YTHDC1 migrate from areas of dispersion in the nucleus to the site of viral RNA synthesis within 18 h of A549 cells infection with AdV5 (Price et al., 2020).

According to the research of Price, the early and late viral transcripts of adenovirus include METTL3-dependent m^6^A modifications, but m^6^A is not necessary for the early phase of adenovirus infection (Price et al., 2020). This group knocked out METTL3 or METTL14 and infected cells 48 h later, and the early gene replication and transcription products of the virus were found to be basically unaffected after infection, but the production of late RNA, late protein, and infectious progeny was significantly reduced in the METTL3- and METTL14-knockout cells (Price et al., 2020). Moreover, the cytoplasmic m^6^A reader and eraser did not affect the adenovirus infection cycle (Price et al., 2020).

The different effects of m^6^A deletion on early and late viral RNAs are mainly due to the important role that m^6^A plays in regulating the splicing efficiency of late viral RNA (Price et al., 2020). Spliced and unspliced viral RNA was detected by qRT-PCR, and the ratio of the two products was used to determine splicing efficiency (Price et al., 2020). After METTL3 or WTAP knockout, the splicing efficiency of the protein encoded by the E1A gene expressed early during infection did not change, while the splicing efficiency of the protein encoded by the fiber gene expressed late in infection decreased significantly (Price et al., 2020). Similar but much less dramatic results were observed upon YTHDC1 knockout (Price et al., 2020). Moreover, METTL3 knockout is widely downregulated during adenovirus late RNA processing. Short-read sequencing results showed that the overall abundance of all viral transcripts in late infection, except that of L152K, was reduced after METTL3 knockout (Price et al., 2020).

#### m^6^A and Respiratory Syncytial Virus

Human respiratory syncytial virus (RSV) is a non-segmented negative strand (NNS) RNA virus in which both genomic and anti-genomic mRNAs undergo m^6^A modification (Whelan et al., 2016). Xue et al. (2019) found that RSV replication was positively regulated by m^6^A reader and writer proteins, but eraser proteins had the opposite, negative regulatory, effect. RT-PCR was used to measure RSV genomic RNA and mRNA in cells. Although overexpression of YTHDF1-3 significantly increased the synthesis of RSV genomic RNA and mRNA, overexpression of YTHDF2 resulted in more active replication of RSV than transcription and more synthesized RNA than mRNA (Xue et al., 2019). When METTL3 and METTL14 were overexpressed in HeLa cells, increased synthesis of viral proteins was observed, and the level of m^6^A marks in viral RNA was significantly higher than that in control groups (Xue et al., 2019). In contrast, eraser proteins downregulated RSV replication and reduced viral RNA m^6^A content (Xue et al., 2019).

Abrogation of m^6^A was shown to result in attenuated RSV infection (Xue et al., 2019). Because m^6^A on the G gene and G mRNA is more abundantly enriched than other genes and mRNA, silent mutation of m^6^A sites in the modified region produced mutated RSV (rgRSVs) expressing recombinant green fluorescent protein (Xue et al., 2019). Compared with parental rgRSV, m^6^A-mutant rgRSVs showed inhibited viral protein synthesis, delayed replication kinetics, and reduced titers (Xue et al., 2019). In addition, in an *in vivo* model of lower respiratory tract infection HAE, replication and spread of m^6^A-site-mutated rgRSVs were defective, and the pathogenicity of these viruses was reduced (Xue et al., 2019). However, the immunogenicity of m^6^A-site-mutant rgRSVs was high in cotton mice (Xue et al., 2019).

#### m^6^A and Human Metapneumovirus

Human metapneumovirus (hMPV) is a NNS RNA virus. Lu et al. (2020) found that the hMPV genome, antigenome and mRNA all contain m^6^A modifications, and the strongest m^6^A peak appeared in the G gene of the genome and antigenome. The effect of m^6^A on hMPV is similar to that on RSV. According to Lu’s research, replication and gene expression of hMPV were promoted by m^6^A modification, and weakening of hMPV infection in cell culture was caused by the abolition of m^6^A (Lu et al., 2020). Both transient and stable overexpression of m^6^A-binding proteins had pro-viral effects in hMPV infection, and the replication, protein and RNA levels of hMPV were significantly upregulated by overexpression of writer proteins (Lu et al., 2020).

#### m^6^A and Influenza A Virus

The influenza A virus (IAV) genome consists of eight single-stranded negative-sense RNA segments that replicate in the nucleus (Noda et al., 2012). Courtney et al. (2017) found that the expression of IAV transcripts was prompted by *cis-*action of m^6^A-modified residues. When METTL3 was knocked out or the m^6^A site in IAV haemagglutinin (HA) was removed, a reduction in viral gene expression, replication and pathogenicity was observed (Courtney et al., 2017). In addition, the expression of the IAV gene and the production of virus particles were increased by the overexpression of YTHDF2, but overexpression of YTHDF1 and YTHDF3 had little effect on IAV replication (Courtney et al., 2017).

According to Courtney’s research, because the deposition of m^6^A not only enhanced splicing but also enhanced the expression of unspliced IAV gene fragments at equivalent levels; therefore, the m^6^A modification is unlikely regulate splicing (Courtney et al., 2017). In addition, the increase in IAV protein expression was closely related to an increase in mRNA expression; the m^6^A modification is also unlikely to enhance translation (Courtney et al., 2017). The mechanism by which the m^6^A modification increases IAV gene expression and viral replication needs to be further explored.

### m^6^A and the Antiviral Response

m^6^A modification not only can affect virus replication but can also regulate the immune response of virus infection, but the influence of the m^6^A modification on the host immune response during virus infection has not been fully elucidated.

Viral RNA genomes and intermediates formed during viral replication often have uncapped RNAs with 5′-triphosphate or 5′-diphosphate groups, which can be detected by cytoplasmic RIG-I (McFadden and Horner, 2020). RIG-I binds non-self RNA and induces signal transduction through mitochondrial antiviral signaling proteins (MAVS) to produce type I and type III interferons (IFNs), thereby activating the antiviral response (McFadden and Horner, 2020). A study by Lu et al. showed that the detection of RNA 5′ triphosphorylation by RIG-I was diminished by the m^6^A modification of the NNS genome and antigenome, and three NNS RNA virus families (*Pneumoviridae*, *Paramyxoviridae*, and *Rhabdoviridae*) imitated host RNA through m^6^A modification to avoid RIG-I recognition (Lu et al., 2021). In another study by Lu, hMPV was used as a model to demonstrate that m^6^A acts as a molecular marker to distinguish self- from non-self RNA via RIG-I (Lu et al., 2020). When the viral antigenome and genomic RNA were deficient in m^6^A marks, enhanced activation of the RIG-I signaling pathway was observed (Lu et al., 2020). In contrast, the demethylase ALKBH5 was recruited by the RNA helicase DDX46 to demethylate m^6^A-modified antiviral transcripts, which were then retained in the nucleus, preventing their translation and inhibiting the production of interferon (Zheng et al., 2017).

Multiple studies have shown that METTL3-mediated methylation of viral RNA m^6^A promotes immune escape. Winkler et al. (2019) demonstrated that the loss of METTL3 led to increased induction of interferon-stimulated immune responses. In addition to METTL3 depletion, the deletion of METTL14 increased IFNB1 mRNA production and reduced viral replication (Rubio et al., 2018). In addition, YTHDF2 deletion led to increased type I interferon levels and greater induction of interferon-stimulated genes (Winkler et al., 2019). YTHDF2 was shown to isolate m^6^A-modified circular RNAs and prevent endogenous circular RNAs from activating the RIG-I antiviral pathway (Lu et al., 2020).

Demethylation of m^6^A viral RNA presents potential antiviral therapeutic opportunities. Methylase inhibitor 3-deazaadenosine (DAA) has an antiviral effect (Gordon et al., 2003; Kennedy et al., 2016; Manners et al., 2019), but because DAA reduces the formation of methyl donor S-adenosylmethionine (SAM) (Fustin et al., 2013) and thus inhibits all types of RNA modification, it is not clear which mechanism of methylation inhibition causes the antiviral effect. Therefore, the development of new drugs specifically targeting the mechanism by which m^6^A modification regulates viral infection will be important to the advancement of antiviral therapy.

## Concluding Remarks

In this review, we discussed the latest developments in the role of m^6^A methylation in the life cycle of respiratory viruses. In AdV, RSV, hMPV, and IAV, m^6^A modification mainly supports virus replication (Table 1). However, in other viruses, such as ZIKV and HCV, m^6^A plays a negative regulatory role in virus replication (Manners et al., 2019). This disparity indicates that m^6^A has a complicated role in the process of viral gene expression.

**TABLE 1:** The regulators of m^6^A in respiratory viruses.

Virus	Molecule	Change	Sample source	Biological function	References
AdV	METTL3/METTL14	Up	A549 cell	Promote viral late gene replication and transcription	Price et al., 2020
	METTL3/WTAP	Up	A549 cell	Promote viral late gene splicing	
	YTHDC1	Up	A549 cell	Promote viral late gene splicing	
RSV	METTL3/METTL14	Up	Hela cell	Promote RSV protein expression	Xue et al., 2019
	YTHDF1-3	Up	Hela cell/A549 cell/Vero cell	Promote RSV replication, mRNA transcription,	
	ALKBH5/FTO	Down	Hela cell	Suppress RSV protein expression	
hMPV	METTL3/METTL14	Up	A549 cell	Promote hMPV replication, viral protein and RNA level	Lu et al., 2020
	YTHDF1-3	Up	A549 cell	Enhance viral protein expression, the release of infectious virus, antigenome and mRNAs	
	ALKBH5/FTO	Down	A549 cell	Suppress virus replication	
IAV	METTL3	Up	A549 cell	Promote IAV republication and virion production	Courtney et al., 2017
	YTHDF2	Up	A549 cell	Promote IAV republication and viral spread	

In addition, m^6^A modification is widely involved in the immune response, but the mechanism by which the m^6^A modification affects antiviral immunity is not fully understood. The role of m^6^A modification in the antiviral immune response needs to be further studied in the future, with the results expected to help in developing new antiviral treatment strategies.

## Author Contributions

QF and HZ wrote the initial draft of the manuscript. ZX reviewed the manuscript. LX proposed the original idea and reviewed the manuscript. This work represents a collaboration among all of the authors. All authors read and approved the final submitted version of the manuscript.

## Conflict of Interest

The authors declare that the research was conducted in the absence of any commercial or financial relationships that could be construed as a potential conflict of interest.

## Publisher’s Note

All claims expressed in this article are solely those of the authors and do not necessarily represent those of their affiliated organizations, or those of the publisher, the editors and the reviewers. Any product that may be evaluated in this article, or claim that may be made by its manufacturer, is not guaranteed or endorsed by the publisher.
